# Conditional Selection of B Cells in Mice With an Inducible B Cell Development

**DOI:** 10.3389/fimmu.2018.01806

**Published:** 2018-08-06

**Authors:** Elias Hobeika, Marcel Dautzenberg, Ella Levit-Zerdoun, Roberta Pelanda, Michael Reth

**Affiliations:** ^1^Centre for Biological Signaling Studies (BIOSS), Biology III, Faculty of Biology, Albert-Ludwigs-University of Freiburg, Freiburg, Germany; ^2^Max Planck Institute of Immunobiology and Epigenetics, Freiburg, Germany; ^3^Department of Molecular Immunology, Biology III, Faculty of Biology, Albert-Ludwigs-University of Freiburg, Freiburg, Germany; ^4^International Max Planck Research School for Molecular and Cellular Biology, Freiburg, Germany; ^5^Department of Immunology and Microbiology, University of Colorado School of Medicine, Aurora, CO, United States

**Keywords:** Cre/l*oxP*, MerCreMer, imb-1 mouse, transitional B cells, Ig-α, tamoxifen, B cell activating factor, B cell development

## Abstract

Developing B cells undergo defined maturation steps in the bone marrow and in the spleen. The timing and the factors that control these differentiation steps are not fully understood. By targeting the B cell-restricted *mb-1* locus to generate an *mb-1* allele that expresses a tamoxifen inducible Cre and another allele in which *mb-*1 expression can be controlled by Cre, we have established a mouse model with an inducible B cell compartment. With these mice, we studied in detail the kinetics of B cell development and the consequence of BCR activation at a defined B cell maturation stage. Contrary to expectations, transitional 1-B cells exposed to anti-IgM reagents *in vivo* did not die but instead developed into transitional 2 (T2)-B cells with upregulated Bcl-2 expression. We show, however, that these T2-B cells had an increased dependency on the B cell survival factor B cell activating factor when compared to non-stimulated B cells. Overall, our findings indicate that the inducible mb-1 mouse strain represents a useful model, which allows studying the signals that control the selection of B cells in greater detail.

## Introduction

B cell lymphopoiesis is a highly regulated process that begins with B cell progenitors (pro-B cells) and progresses through distinct stages (1–3). Pro-B cells, like B cells at later stages, are distinguished by the expression of Igα and Igβ, the respective products of the B cell-specific genes *mb-1* and *B29* (2). These two proteins are crucial for B cell development. Indeed, the loss of Igα or Igβ expression in knockout mice (4–6), or in rare cases of human Igα or Igβ deficiency (7–9), results in a complete block of B cell development at the pro-B cell stage. This is because the developmental progression of pro-B cells requires the expression of the precursor B cell antigen receptor (pre-BCR) (10, 11) which comprises the μm heavy (H) chain, a surrogate light chain (composed of VpreB and lambda 5 chains), and the Igα/Igβ (CD79a/CD79b) heterodimer (12). Upon the expression of a functional pre-BCR, the pre-B cells first proliferate, then rearrange their Ig light chain loci and differentiate into immature B cells carrying a B cell antigen receptor (BCR) of the IgM class on their surface (13, 14). The immature B cells leave the bone marrow (BM) to continue their differentiation in the spleen (15–19).

The IgM-expressing immature B cells in the spleen are divided into two major subgroups, namely the transitional 1 (T1) and transitional 2 (T2) B cells (20, 21). T1-B cells are negative for the surface markers CD23 and CD21 whereas T2-B cells express both markers (21, 22). A third transitional population, T3-B cells have been described. They arise from T2 B cells and have a similar phenotype, with the exception of IgM expression, which is strongly down modulated (20). However, T3-B cells are believed to represent an unresponsive (anergic) state rather than an intermediate maturation stage (23, 24). All transitional B cells also express the CD93 (AA4.1) marker originally detected by a monoclonal antibody (clone 493) generated by the Rolink group (22). The T2-B cells then develop into CD93 (AA4.1)^−^ mature follicular (M) and marginal zone (MZ) B cells defined as IgM^low^IgD^high^CD23^high^CD21^+^ and IgM^high^ IgD^low^CD23^low^CD21^high^ cells, respectively (13, 20, 21, 25). Both cell fates are controlled by BCR-mediated signaling pathways (21, 26, 27).

The further development of T2-B cells requires the B cell activating factor (BAFF) (28–33), which is also known as Blys, and signaling through the classical and alternative NF-κB pathways (34–36). BAFF is a member of the TNF family and is implicated in peripheral B cell development. Mice lacking the BAFF-receptor (BAFF-R or BR3) have a block at the T1 stage (37, 38). On the other hand, mice overexpressing BAFF have a lenient peripheral B cell selection and develop autoimmune diseases (39, 40).

Cre is a site-directed DNA recombinase that specifically cuts DNA at *loxP* sites and can be employed for the activation or deletion of genes in the mouse (41–44). Previously, we and others have shown that chimeric Cre proteins with an appended mutated binding domain of the murine α-estrogen receptor (Mer) can be regulated by tamoxifen (45–48). In particular, MerCreMer, a fusion protein carrying a Mer domain at both the N- and C-terminus of Cre, demonstrates a very tight regulation of recombinase activity (49). This construct has been prominently used to study heart muscle development and hematopoietic stem cell fates (50–52). In the past, we have used a related inducible Cre system to study mature B cells lacking the expression of the spleen tyrosine kinase Syk or that of Igα and the BCR (53, 54). Here, we employ the MerCreMer/*loxP* system to generate mice in which the expression of the *mb-1* gene, and thus of Igα, is induced by tamoxifen treatment. With this system, we can generate a short wave of developing B cells in the adult mouse and monitor the kinetics of their development. At day 5 post induction (p.i.) most B cells in these mice are transitional T1-B cells, which are thought to be highly sensitive to negative selection upon BCR engagement (55, 56). Surprisingly, the *in vivo* stimulation of the T1-B cells with anti-IgM antibodies does not lead to their deletion but rather their survival and accelerated differentiation to the T2-B cell stage by upregulation of Bcl-2. The survival of stimulated T2-B cells requires, however, the presence of BAFF or the BAFF-R.

## Results

### Generation of Mice With an Inducible B Cell Development

**Figure 1:**
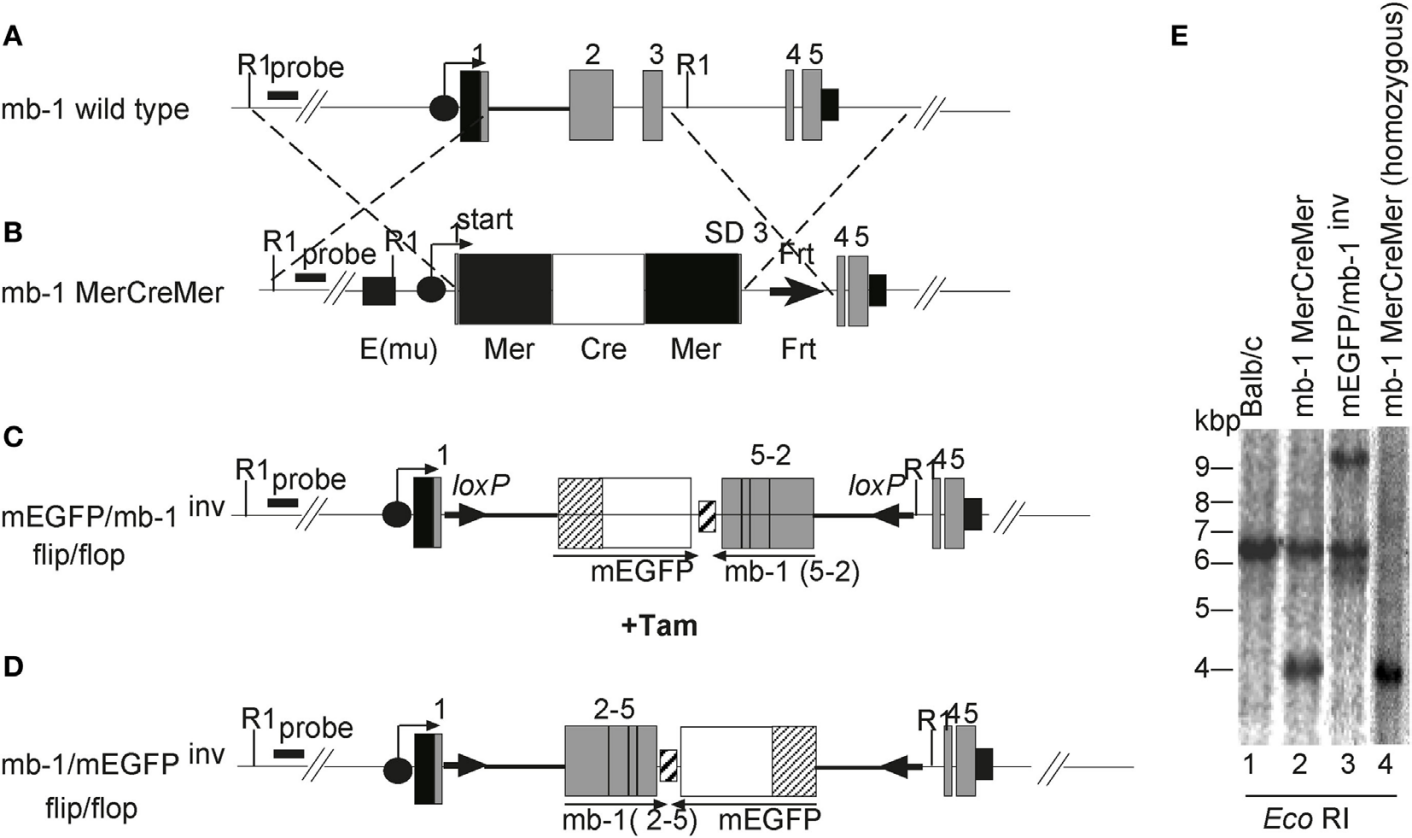
Generation of mice carrying a modification at the *mb-1* locus. Schematic representations of **(A)** the *mb-1* wild-type allele and the knockin alleles, **(B)** mb-1 MerCreMer, **(C)** membrane-bound form of the enhanced green fluorescent protein (mEGFP)/mb-1^inv^, and **(D)** mb-1/mEGFP^inv^ (not drawn to scale). The mb-1MerCreMer strain was crossed to either mEGFP/mb-1^inv^ or mb-1/mEGFP^inv^ to generate the imb-1 and the cmb-1 strains, respectively. The black circles with an arrow represent the *mb-1* promoter. The gray boxes indicate the *mb-1* exons, and the small black box after exon 5 represents the 3′ untranslated region and the *mb-1* polyA sequence. In **(B)**, the black square 5′ of the *mb-1* promoter represents the immunoglobulin heavy chain intron enhancer (Emu), the black and white boxes represent (Mer) and the humanized Cre (hCre), respectively. The black triangle indicates the Frt site left after deletion of the Neo^r^ cassette by the transient transfection with the enhanced flp recombinase (flpe). Constructs **(C,D)** were previously described in Ref. (47). **(E)** Southern blot analysis of mEGFP/mb-1^inv^ and mb-1 MerCreMer heterozygous mice relative to wild-type control. Genomic DNA was prepared from tails of the designated mice, digested with *Eco*RI and hybridized with a radioactive labeled 5′ external probe to identify the homologously recombined alleles. The analysis by Southern blot gives 4.1 and 9.6 kb fragments for the DNA targeted with MerCreMer and the “flip/flop” cassette (mEGFP/mb-1^inv^ or mb-1/mEGFP^inv^), respectively (Figure 1E, lanes 2 and 3 and data not shown). Wild-type DNA generates a 6.4 kb fragment with the same enzyme (Figure 1E, lane 1). Lane 4 represents an mb-1 MerCreMer homozygous mouse.

**Figure 2:**
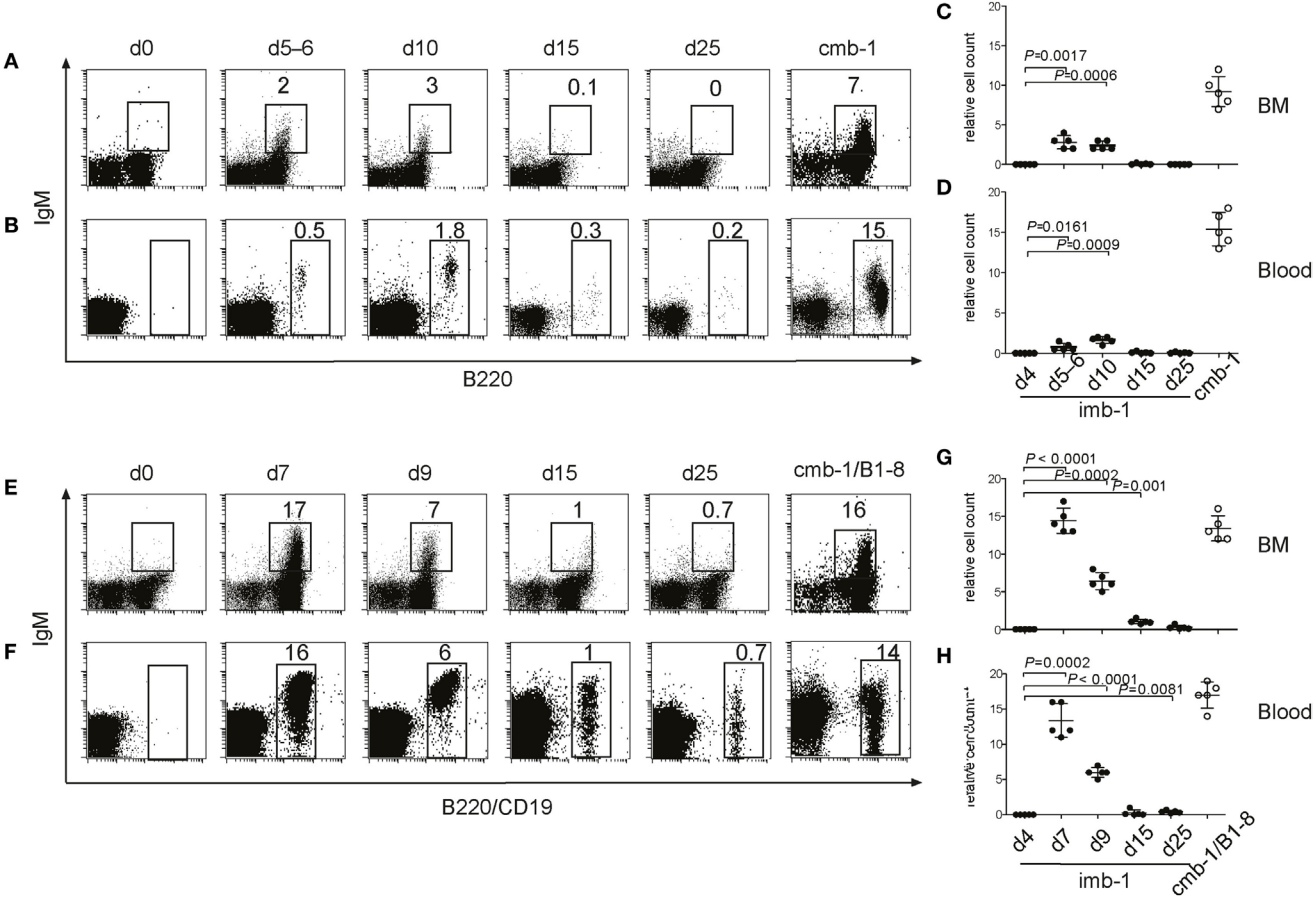
B-cell migration from the bone marrow (BM) into the secondary lymphoid organs of imb-1 mice takes place in a wave. **(A,B)** B220 versus IgM staining of total lymphocytes derived from BM **(A)** or blood **(B)** of imb-1 mice analyzed at different time points after a single treatment with tamoxifen [days 5–6, 10, 15, and 25 post induction (p.i.)]. Uninduced mice (d0) as well as cmb-1 mice served as controls. Representative data from three independent experiments with two mice at each time point are shown. **(C,D)** Statistical analysis of the relative cell count (percentage) of B220^+^IgM^+^ B cells (BM) or total B220^+^ B cells (blood) in the total lymphocyte gate of tamoxifen-treated imb-1 mice at days 4, 5–6, 10, 15, and 25. Mean ± SD of *n* = 5 is indicated for each time point; each dot represents an individual mouse. *P* values are indicated and were obtained using the one-sample *t*-test. **(E,F)** IgM versus B220 (upper panel; BM), or IgM versus CD19 (lower panel; blood) staining of total lymphocytes derived from the BM **(E)** and blood **(F)**, respectively, of imb-1/B1-8H (designated imb-1/B1-8) mice at indicated time points p.i. Uninduced mice (d0) as well as cmb-1/B1-8H mice served as controls. The percentages for B220^+^ IgM^+^ (BM) and total CD19^+^ (blood) cells inside each box are indicated. Representative data from two independent experiments with two to three mice at each time point are shown. **(G,H)** Statistical analysis of the percentage of B220^+^IgM^+^ B cells (BM) or CD19^+^ B cells (blood) within the lymphocyte gate in tamoxifen-treated imb-1/B1-8H mice at days 4, 7, 9, 15, and 25. Mean ± SD of *n* = 5 is indicated for each time point; each dot represents an individual mouse. *P* values are indicated and were obtained using the one-sample *t*-test.

To study the kinetics of B cell development *in vivo*, we generated mice that are born without mature B cells but that can be induced to transiently produce B cells at any time in their life. In these mice the expression of the *mb-1* gene, which is essential for B cell development, can be regulated by our MerCreMer/*loxP* technique. The *mb-1* gene has five exons (Figure 1A). Using BALB/c embryonic stem (ES) cells, this gene was altered by homologous recombination with two different targeting vectors to create two distinct mutant alleles. The first vector was used to replace *mb-1* exons 1, 2, and 3 with a cDNA sequence encoding MerCreMer (Figure 1B). The MerCreMer cDNA cassette is therefore transcribed under the control of the endogenous *mb-1* promoter, further enhanced through the insertion of the μ intronic enhancer inserted 5′of the *mb-1* promoter. In the second targeting experiment, which we have previously described (47), we inserted a “flip/flop” mb-1/EGFP cassette flanked by two *loxP* sites (“floxed”) pointing in opposite directions (Figure 1C). This cassette, which replaced *mb-1* exons 2 and 3, contained a cDNA coding for a membrane-bound form of the enhanced green fluorescent protein (mEGFP) and, in the opposite transcriptional orientation, a cDNA of the *mb-1* coding sequence from exon 2 through exon 5 (Figure 1C). The mEGFP/mb-1^inv^ allele produces mEGFP in place of Igα, which, in the absence of another *mb-1* functional allele, leads to the arrest of B cell development at the pro-B cell stage. Cre-mediated recombination can invert the floxed DNA sequence, thus placing the *mb-1* exons 2–5 cDNA in the right orientation to allow the proper splicing from exon 1 (Figure 1D). This newly generated mb-1/mEGFP^inv^ allele expresses Ig-α (but not EGFP) allowing for B cell generation. Thus, the mb-1 “flip/flop” allele can mediate the alternative expression of either Ig-α or mEGFP. Germ line transmission of the two targeted *mb-1* alleles (mb-1MerCreMer and mEGFP/mb-1^inv^) were confirmed by Southern blot analysis of tail genomic DNA (Figure 1E). We then generated BALB/c mice that carry both differently targeted *mb-1* alleles and thus are defective in Ig-α expression. Here, we refer to these mice with a tamoxifen inducible (i) switch from EGFP to Ig-α expression as imb-1 mice. In the BM, these mice have B220^+^ pro-B cells but no IgM^+^ B cells (Figure 2A, d0). B cells are also absent from the blood (Figure 2B, d0). The fact that the imb-1 mice display a complete block of B cell development at the pro-B cell stage is also demonstrated by the absence of immunoglobulin isotypes in their sera (data not shown). These data show overall that MerCreMer activity is tightly regulated in the mouse.

### Kinetics of B Cell Development in the BM and Spleen of imb-1 Mice

To efficiently activate MerCreMer in pro-B cells, we treated the imb-1 mice orally with a single dose of 6 mg of tamoxifen citrate dissolved in lipids. Already 4 h after this treatment, the mice contained enough tamoxifen in the blood to activate MerCreMer in a cultured B cell line carrying an EGFP reporter cassette, and sufficient levels of tamoxifen were maintained until day 4 after administration (Figure S1 in Supplementary Material). In the BM and the blood of tamoxifen-treated imb-1 mice, IgM^+^ B cells were first detected between days 5 and 6 p.i. and reached their highest levels at day 10 p.i. (Figures 2A–D). The late onset of IgM^+^ B cell appearance in the treated imb-1 mice may be due to the fact that the mEGFP/mb-1^inv^ allele only becomes stabilized once the tamoxifen concentration and MerCreMer activity has declined (days 5–6; Figure S1A in Supplementary Material), in addition to the need for productive rearrangements at both the Ig heavy and light chain loci. After day 15, only very few IgM^+^ B cells were observed in the BM and blood of treated mice (Figures 2A–D, d15). Apparently at this time point, all newly generated B cells have left the BM and, in the absence of (tamoxifen and) MerCreMer activity, new B cells cannot be generated. Thus, only a short wave of B cell development is induced in tamoxifen-treated imb-1 mice. As a positive control for these experiments, we analyzed the BM and blood of untreated mice carrying in the germ line the mb-1/mEGFP^inv^ and mb-1MerCreMer alleles (cmb-1 mice) and that have, therefore, a continuous mb-1 expression and unperturbed B cell development (Figures 2A–D).

To assess factors influencing the efficiency of B cell production, the imb-1 and the cmb-1 control mice were bred with B1-8H mice (57), which carry the productively rearranged V_H_D_H_J_H_ exon coding for the B1-8 VH domain as a knockin at the *Igh* locus. All pro-B cells of imb-1/B1-8H and cmb-1/B1-8H mice express a μ-chain in the cytoplasm (data not shown). In spite of the early H chain expression in all pro-B cells, no B cell development took place beyond this stage in untreated imb-1/B1-8H mice, again demonstrating the tight regulation of the MerCreMer enzyme *in vivo* (Figures 2E,F, d0). Upon treatment with tamoxifen, the imb-1/B1-8H mice produced threefold to fivefold more B cells in the BM and the blood than imb-1 mice, although with similar kinetics of B cell development (Figures 2C–H). These data suggest that the major factor responsible for delayed B cell development in imb-1 mice is the rate of tamoxifen catabolism and the consequent stabilization of the inducible *mb-1* allele.

**Figure 3:**
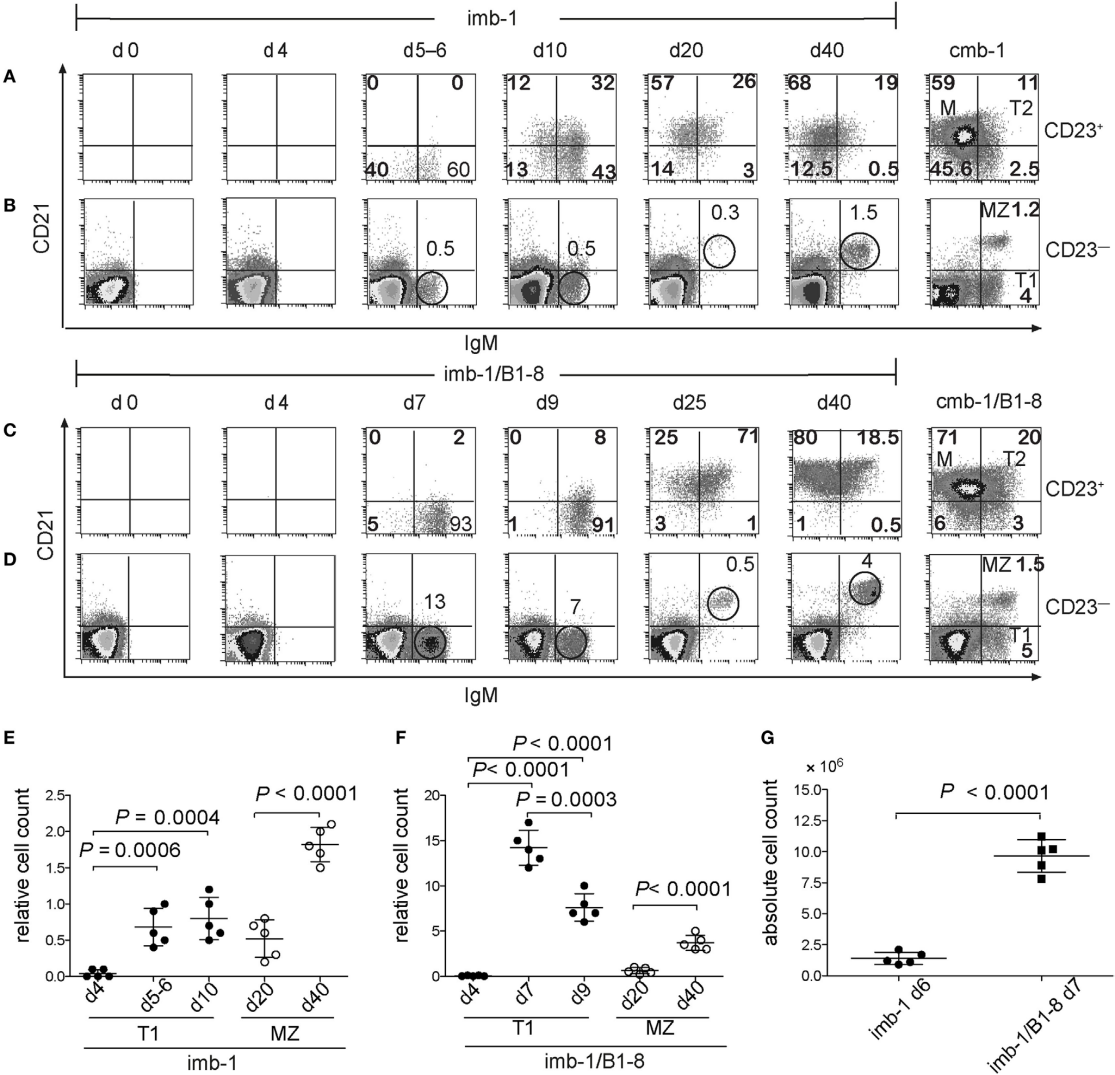
Transitional B cells express CD23 before CD21. **(A)** FACS analysis of CD21 and IgM expression on the CD23^+^ **(A,C)** and CD23^−^ **(B,D)** lymphocyte populations derived from the spleen of mice before (d0) or at indicated time points after a single treatment with tamoxifen. Cmb-1 and cmb-1/B1-8 mice with a constitutive B-cell development were analyzed in parallel. The percentages of the transitional 1 (T1) and marginal zone (MZ) populations within the CD23^−^ lymphoid gate at the designated time points post induction are indicated above the corresponding circle. Within the CD23^+^ lymphoid gate, the percentages of the transitional 2 (T2) and M populations are indicated within the upper quadrants. Representative data from two independent experiments with total five mice at each time point are shown. **(E,F)** Quantitative analysis of relative T1 and MZ-B cell numbers (percentage of cells within the CD23^−^ lymphoid gate) in imb-1 or imb-1/B1-8H mice at designated time points; mean ± SD of *n* = 5 is indicated for each time point; each dot represents an individual mouse; *P* values were determined using a two-tailed Student’s *t*-test and are indicated in the graphs. **(G)** Absolute B cell numbers in the spleens of imb-1 mice and imb-1/B1-8H mice at days 6 and 7, respectively; mean ± SD of *n* = 5 is indicated for each time point; each dot represents an individual mouse; *P* values were determined using a two-tailed Student’s *t*-test and are indicated in the graphs.

In the spleens of tamoxifen-treated imb-1 mice, the first immigrant B cells were detected at days 5–6 p.i. (Figures 3A,B,E). These B cells expressed IgM and were mostly negative for CD21 and CD23, thus displaying the phenotype of T1-B cells (Figures 3A,B,E, d5–d6). Some B cells were detected within the CD23^+^ gate at days 5–6, which had not yet acquired CD21 expression (Figure 3A). By day 10, roughly 56% of the B cells within the CD23^+^ gate were IgM^dim/+^, but still negative for CD21 whereas the other 44% were IgM^dim/+^ CD21^+^, thus displaying the phenotype of T2-B cells (Figures 3A,B, d10). Therefore, during their maturation in the spleen, B cells seem to first acquire CD23 and only later the CD21 surface marker. Small amounts of mature follicular B cells (CD23^+^CD21^+^and IgM^low^) appeared at day 10 and were the majority of splenic B cells by day 20 p.i. (Figure 3A, d10 and d20). Mature B cells persisted in the spleen of induced imb-1 mice for 40 days (Figure 3, d40), although their numbers declined with time (data not shown).

Marginal zone B cells (IgM^high^CD21^+^ but CD23^−^) were first detected at day 20 p.i. and became more prominent by day 40 p.i. (Figures 3B,E; d20 and d40). This late development of MZ B cells is in accordance with their time of appearance in neonates and immune deficient mice after adoptive transfer (21, 58). The kinetics of B cell development in the spleen of imb-1/B1-8H mice was similar to that seen in imb-1 mice, although higher numbers of B cells were generated (Figures 3C,D,F,G). The improved cellular yields in imb-1/B1-8H mice show even more clearly that B cells acquire the CD23 marker prior to CD21 expression (Figure 3C, d7–d25; Figure S1B in Supplementary Material; d7).

### No Deletion of T1-B Cells Upon *In Vivo* Treatment With Anti-IgM

Immature T1-B cells in the spleen are characterized as CD93 (AA4.1^+^), CD23^−^, CD21^−^, IgM^high^, and IgD^−/low^ (20, 21). Based on *in vitro* studies, the T1-B cells have been considered to be sensitive to negative selection and to undergo apoptosis upon engagement of their BCR (59). The imb-1/B1-8H mice provide an ideal system to test this hypothesis *in vivo*. Whereas in the spleens of wild-type mice T1-B cells are a minority, these cells are the majority of B cells in the spleen of imb-1/B1-8H mice at days 5–6 after their treatment with tamoxifen (Figure S2 in Supplementary Material). Furthermore, at days 5–6 p.i., imb-1/B1-8H mice have not produced any soluble antibodies (data not shown) and, thus, injected anti-IgM reagents can bind to the BCR on the surface of T1-B cells without being quenched by soluble IgM in the serum.

**Figure 4:**
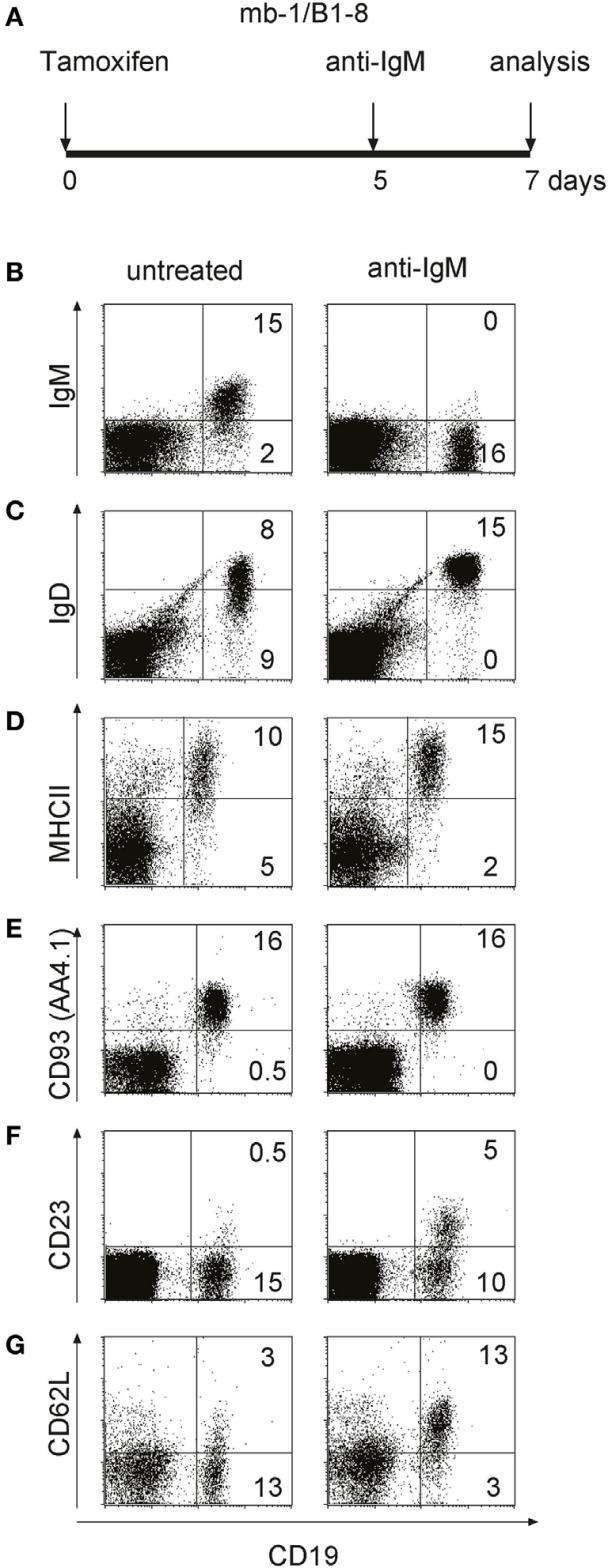
*In vivo* activation and maturation of anti-IgM treated transitional 1 (T1)-B cells. **(A)** Imb-1/B1-8 mice were treated with F(ab′)_2_ fragments of anti-mouse IgM at day 5 post induction (p.i.) and lymphocytes derived from the spleen were analyzed 2 days after treatment. **(B–G)** Splenic lymphocytes of untreated (left panel) and anti-IgM treated (right panel) imb-1/B1-8 mice at d7 p.i. were isolated and stained with antibodies against CD19 in combination with those against MHC class II, IgD, IgM, CD93 (AA4.1), CD23, or CD62L. Representative data from six independent experiments with two mice are shown.

To test for the behavior of T1-B cells upon engagement of their BCR *in vivo*, we treated imb-1/B1-8H mice at late day 5 p.i. (when the newly generated B cells first appeared in the blood of most mice) with 100 µg of anti-IgM F(ab′)_2_ and analyzed their spleens at day 7 p.i. by flow cytometry (Figure 4A). Compared to B cells in untreated mice, the T1-B cells in anti-IgM treated imb-1/B1-8H mice appeared activated and/or differentiated as indicated by higher MHC class II and IgD-BCR expression, but reduced IgM-BCR levels on their cell surface (Figures 4A–D). Since the anti-IgM treatment leads to IgM-BCR internalization (data not shown) and the increase of IgD may not be a good indicator of further maturation, we assessed B cells of imb-1/B1-8H mice for additional surface markers that identify the stage of B cell maturation. While the T1-B cells of tamoxifen-treated mice still expressed CD93 (AA4.1: Figure 4E), which identifies a transitional stage, they also displayed the CD23 and CD62L markers, suggesting that upon stimulation they undergo an enhanced differentiation toward the T2-B cell stage (Figures 4F,G) (21, 59). For these experiments, we chose mice, which at day 5 p.i. had similar amounts of B cells in their blood. As the B cell numbers in the spleens of treated imb-1 mice could vary from mouse to mouse, we repeated the experiment several times using imb-1/B1-8H and imb-1 mice. In all experiments, anti-IgM treatment led to an increased differentiation toward the (IgD^high^IgM^low^) T2-B cell stage, but not to a reduction of absolute B cell numbers in the spleen (Figure S3A in Supplementary Material). This was also confirmed by a transfer experiment where we isolated EGFP^+^CD23^−^ T1-B cells from induced RERT/EGFP mice (described in Figures S4D,E in Supplementary Material) at day 5 p.i. and injected the same number of these B cells into Rag2^−/−^γ_c_^−/−^ mice. Three hours after transfer, the host mice were either left untreated or treated with 100 µg of anti-IgM F(ab′)_2_ and the B cells of these mice were analyzed 36 h later by flow cytometry (Figure S3B in Supplementary Material). Again, *in vivo* stimulated T1-B cells showed an increased differentiation to T2-B cells (IgD^high^IgM^low^ and CD23^+^), but the B cell numbers were similar in the untreated and anti-IgM treated mice (Figure S3 in Supplementary Material and data not shown).

Overall, these data indicate that transitional B cells undergo activation and differentiation and not death upon their BCR engagement *in vivo*.

### Stimulated T2-B Cells Require a BAFF Signal for Survival

**Figure 5:**
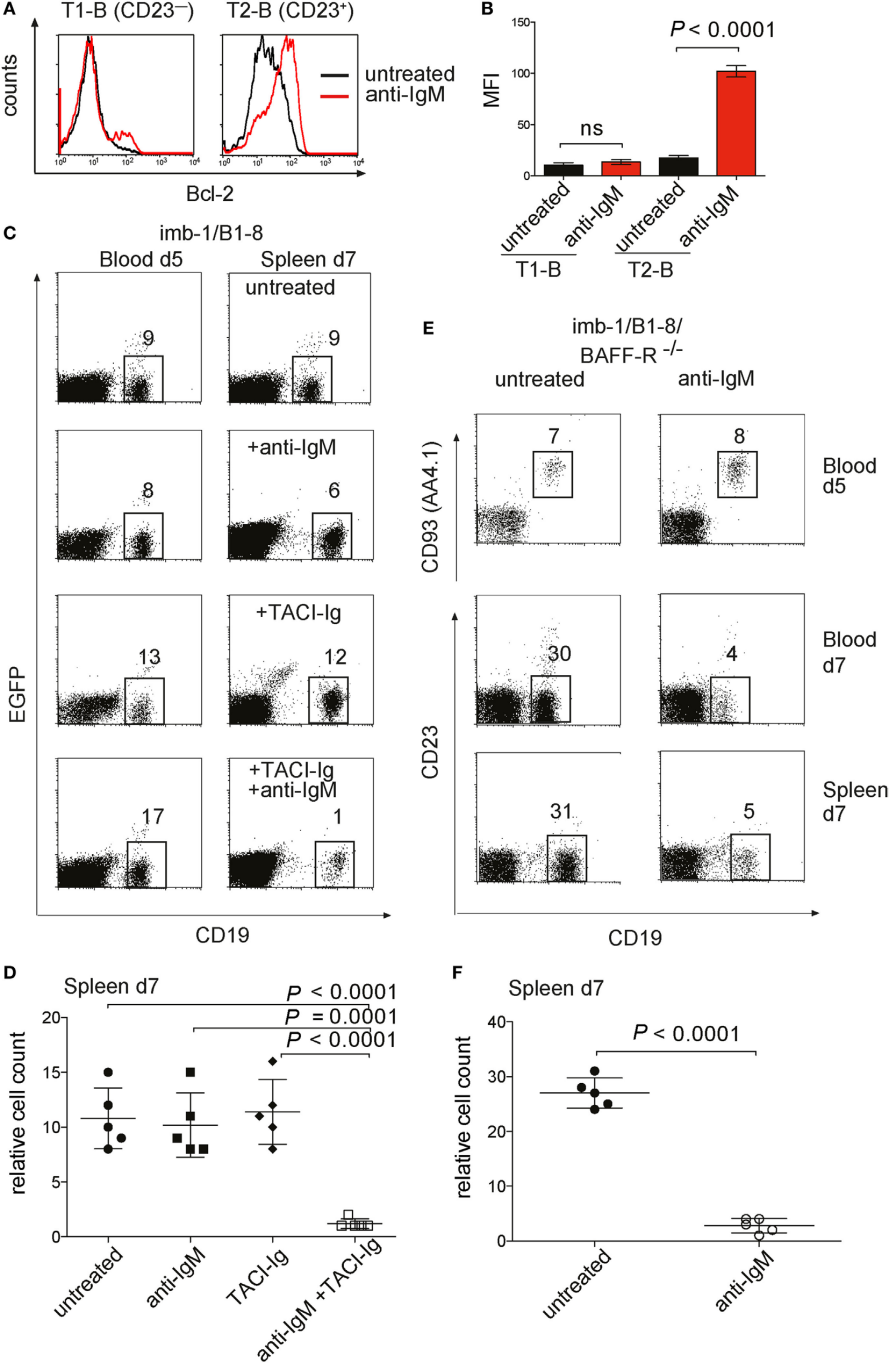
Bcl-2 expression and survival of transitional B cells from anti-IgM treated imb-1. **(A)** Histograms of intracellular expression level of Bcl-2 in splenic B cells derived from CD21^−^CD23^−^IgM^high^IgD^low^ transitional 1 (T1) (left panel) and CD21^+^CD23^+^IgM^high^IgD^high^ transitional 2 (T2) (right panel) B cells. B cells from untreated mice, black line; from anti-IgM treated mice, red line. The cells were analyzed at day 7 post induction (p.i.) (2 days after anti-IgM injection). Representative data from five independent experiments with two mice are shown. **(B)** Statistical analysis of median fluorescence intensity (MFI) values of Bcl-2 in the designated B cell population with or without anti-IgM treatment. Mean ± SD of *n* = 5 is indicated for each time point; The *P* value was obtained using a two-tailed Student’s *t*-test. **(C)** Treatment of imb-1/B1-8H derived splenocytes with TACI-Ig and anti-IgM. Left panel: CD19 versus EGFP dot plots of lymphocytes from the blood of imb-1/B1-8 mice at day 5 p.i. Right panel: CD19 versus EGFP dot plot of lymphocytes from spleens at day 7 p.i. treated with anti-IgM (upper row), TACI-Ig (middle row), or a combination of anti-IgM and TACI-Ig (bottom row). The numbers in gates indicate percentages of B cells. Representative data from three independent experiments with two mice for each treatment are shown. **(D)** Quantitative analyses of imb-1/B1-8H-derived splenic CD19^+^ B cells (within lymphoid gate) at day 7 p.i. Treatment is designated in the graph; mean ± SD of *n* = 5 is indicated for each time point; each dot represents an individual mouse; *P* values were obtained using a two-tailed Student’s *t*-test and are indicated in the graphs. **(E)** Anti-IgM treatment of transitional B cells in the absence of BAFF-R. imb-1/B1-8/BAFF-R^−/−^ mice at day 5 p.i. were either left untreated (left panel) or treated with anti-IgM (right panel). Upper row: CD19 versus CD93 (AA4.1) dot plot on blood derived B cells at day 5 p.i. before anti-IgM injection. The middle and lower rows depict dot plot analyses representing CD19 versus CD23 expression on B cells derived from either blood (middle row) or spleen (bottom row) at day 7 p.i., which were left untreated (left panel) or received anti-IgM at day 5 p.i. (right panel). Representative data from three independent experiments with two mice are shown. **(F)** Quantitative analyses of imb-1/B1-8H/BAFF-R^−/−^-derived splenic B cells (%CD19^+^ in lymphoid gate) at day 7 p.i. Treatment is designated in the graph; mean ± SD of *n* = 5 is indicated for each time point; each dot represents an individual mouse; *P* value was obtained using a two-tailed Student’s *t*-test and is indicated in the graphs.

To elucidate the molecular mechanism behind the surprising survival of *in vivo* stimulated T1- and T2-B cells, we analyzed the expression of anti-apoptotic molecules in these cells. In particular, the pro-survival factor Bcl-2 plays an important role in the survival of B cells at different developmental stages. To test for Bcl-2 expression in T1- and T2-B cells, we used specific anti-Bcl-2 antibodies and an intracellular staining protocol. Interestingly, T2-B cells isolated at day 7 p.i. from an anti-IgM-treated imb-1/B1-8H mouse showed elevated Bcl-2 levels in comparison to B cells isolated from untreated mice (Figure 5A, right panel, and Figure 5B). To exclude that the observed differences in Bcl-2 levels were due to an increased cell size after anti-IgM treatment, we assessed the forward scatter (FSC), which is directly proportional to cell size, of the T2 B cells derived from either untreated or anti-IgM-treated mice. We found no increase in cell size in T2 B cells from anti-IgM-treated mice. In fact, the FSC of these cells was somewhat reduced compared to the untreated control cells (Figure S4A in Supplementary Material). Notably, no difference in Bcl-2 expression level was found in T1-B cells isolated from either untreated or anti-IgM-treated mice (Figure 5A, left panel, and Figure 5B). A previous study has proposed that T1-B cells are lost whereas T2-B cells persist upon BCR engagement due to expression of the pro-survival factor Bcl-xL (60). We have assessed the expression of Bcl-xL in T1- and T2-B cells (separated based on CD23 expression) and found no induction of Bcl-xL upon anti-IgM treatment (Figure S4B in Supplementary Material). These data suggest that the engagement of the BCR on T1-B cells not only promotes differentiation to the T2-B cell stage but also initiates a survival program by upregulation of Bcl-2.

The transcription of the Bcl-2 gene is under the control of the NF-κB pathway (61), and it has been shown that signals from the BCR and the BAFF-R cooperate in NF-κB activation (62). The spleen of imb-1 and imb-1/B1-8H mice contains no B cells before tamoxifen treatment and only a few mature B cells are detected in the spleens at days 5–7 after induction (Figure 3G). Therefore, one can assume that there is little competition among these B cells for the abundant BAFF in the spleen of these mice. This notion is supported by previous publications reporting that soluble BAFF levels rise in the absence of B cells and excessive BAFF concentrations lead to increased survival of antigen-engaged B cells (63–67). To study the role of BAFF in the survival and differentiation of B cells in the imb-1 mouse, we interfered with BAFF signaling by either removing BAFF with the soluble decoy receptor TACI-Ig or by employing BAFF-R-deficient mice (38). We injected imb-1/B1-8H mice with anti-IgM, TACI-Ig (at days 0, 3, and 5 p.i.), or both reagents and monitored the number of CD19^+^ B cells in the blood at day 5 after the final TACI-Ig and before anti-IgM injection, and at day 7 after treatment in the spleen (Figures 5C,D). Whereas each reagent alone had no effect on B cell numbers, the combination of both reagents resulted in a drastic reduction of B cells (from 10.8 ± 2.8% in the untreated control to 1.2 ± 0.4% after TACI-Ig + anti-IgM treatment at day 7, Figures 5C,D) in the spleen. These data indicate that the BCR-stimulated T1-B cells require BAFF signaling for their survival whereas naive T1-B cells can survive in the spleen for some time without BAFF.

A similar conclusion was drawn from the study of imb-1 mice lacking the BAFF-R. After the induction of B cell development, the imb-1/B1-8H/BAFF-R^−/−^ mice were either left untreated or received anti-IgM F(ab′)_2_ at day 5 p.i. (Figures 5E,F). In the absence of BCR stimulation, the BAFF-R-deficient T1-B cells developed normally and were first detected in the blood at day 5 p.i. However, the treatment with anti-IgM resulted in a drastic reduction of B cell numbers at day 7 p.i. in the blood (from 30 to 4%) and in the spleen (from 27 ± 2.7 to 2.8 ± 1.3%) in these mice (Figures 5E,F). Furthermore, B-cell development in the imb-1/BAFF-R^−/−^ mice is partially blocked at the T1-B cell to T2 transition, which is evident by the lack of CD23 marker characteristic for T2-B cells (Figure 5E, lower panel, relative to Figure 4F).

The imb-1 mice have only one functional but targeted *mb-1* allele (mb-1/mEGFP^inv^) and therefore their T1-B cells express Ig-α and the IgM-BCR at twofold to threefold lower amounts than T1-B cells from wild-type mice [Figure S4C in Supplementary Material and Ref. (68)]. To exclude that the lack of negative selection of T1-B cells in anti-IgM treated imb-1 mice is due to the lower IgM-BCR expression and thus signaling (68, 69) we developed another mouse model (RERT/EGFP) with an inducible B cell compartment. RERT mice express tamoxifen-regulated Cre-ER^T2^ under the control of the RNA polymerase II promoter (70, 71). RERT mice were crossed to mEGFP/mb-1^inv^ mice to generate RERT/EGFP animals that carry two floxed mEGFP/mb-1^inv^ alleles (Figure S4D in Supplementary Material). A Cre-ER^T2^-mediated inversion of both *mb-1* alleles results in the loss of EGFP, allowing the expression of two copies of *mb-1*. This leads to higher IgM-BCR surface level in RERT/EGFP T1-B cells compared to imb-1-derived T1-B cells (Figures S4E,F in Supplementary Material). The kinetics of B cell development and the upregulation of Bcl-2 in anti-IgM treated T2-B cells were similar in RERT/EGFP and imb-1 mice (Figure S4G in Supplementary Material and data not shown). Therefore, the survival of transitional B cells in anti-IgM treated imb-1 mice is not the result of low IgM-BCR expression but rather a part of their normal developmental program in the presence of excess BAFF.

**Figure 6:**
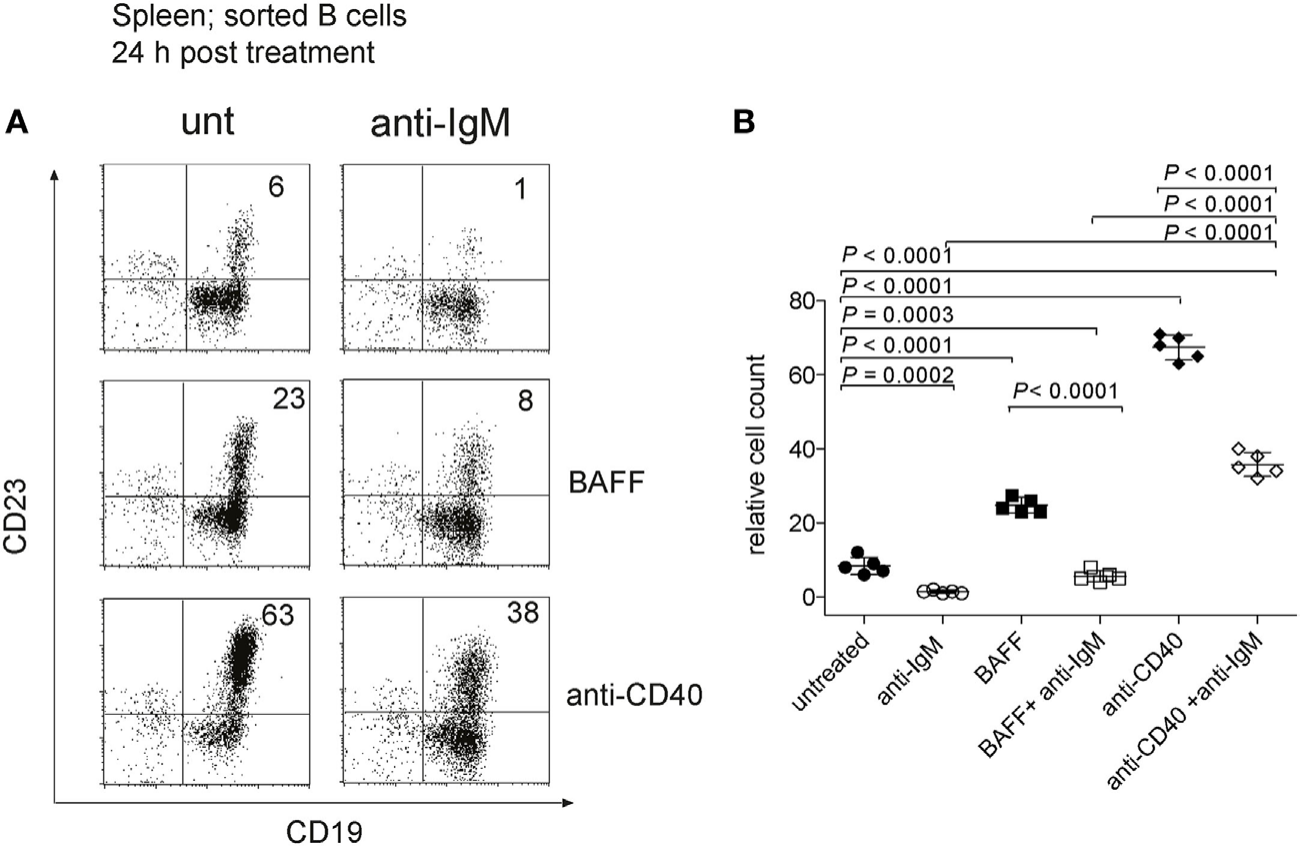
Anti-IgM treatment of transitional 1 (T1) B cells *in vitro*. **(A)** To obtain T1-B cells, imb-1-derived splenocytes were sorted at day 6 post induction using CD43 and Thy1.2 antibodies to exclude non-B cells. Identical cell numbers were cultured *in vitro* in the absence or presence of anti-IgM (10 µg/ml) and/or human recombinant B cell activating factor (BAFF) (75 ng/ml) and anti-CD40 antibodies (10 µg/ml) for 24 h. Expression of CD19 and CD23 is shown. The cells were stained with 7AAD to exclude dead cells and only viable 7AAD cells were included in the analysis. The numbers in quadrants indicate relative cell numbers acquired after analysis of 10^5^ total events per sample. Representative data from three independent experiments with two duplicates for each treatment are shown. **(B)** Quantification of relative cell numbers form the experiment depicted in **(A)**. Stimuli are indicated in the graph. *P* values were obtained using a two-tailed Student’s *t*-test and are indicated; Mean ± SD values are indicated for *n* = 5. Each dot represents an individual replicate.

Previous studies have shown that immature B cells stimulated with anti-IgM *in vitro* die rapidly by apoptosis (55), whereas we found survival of these cells *in vivo*. To clarify this issue, we isolated T1-B cells from imb-1 mice at day 5 p.i. and cultured them *in vitro* for 24 and 48 h without or with 10 µg/ml of anti-IgM F(ab′)_2_. During the 24 h *in vitro* culture, approximately 6% of T1-B cells developed into CD23^+^ B cells (Figures 6A,B). The anti-IgM treatment greatly reduced the numbers of these cells (Figures 6A,B). We cultured the isolated T1-B cells either with BAFF (75 ng/ml) or anti-CD40 (10 µg/ml) antibody, two stimuli that activate the NF-κB pathway in B cells (72). In the presence of these reagents, 23 and 63% of the living B cells, respectively, displayed CD23 expression (Figures 6A,B). The increase of CD23^+^ cells in the BAFF containing culture may be a result of CD23 gene activation *via* BAFF, which has been described before (73). Anti-CD40 treatment resulted in a very high expression of CD23 indicating that the cells indeed differentiated, possibly in T2 B cells (Figures 6A,B). It is feasible that longer stimulation with anti-CD40 might lead to differentiation of these cells into blasts as they become more mature (74). The addition of either BAFF or anti-CD40 only partially prevented the cultured T1-B and T2-B cells from anti-IgM-mediated apoptosis after 24 h (Figures 6A,B). However, only 1, 3, and 6% of viable cells were detected after 48 h of culture stimulated with anti-IgM alone or in combination with BAFF or anti-CD40, respectively (Figure S5 in Supplementary Material). These results suggest that upon BCR engagement, T1-B cells require additional factors apart from BAFF and CD40 for their survival and differentiation *in vivo*.

## Discussion

In this study, we show that the tamoxifen-induced MerCreMer recombinase is tightly regulated *in vivo* and can be used to activate genes in the mouse. We took advantage of this feature to generate the imb-1 mouse with an inducible B cell compartment. Only upon tamoxifen-treatment do imb-1 mice produce mature B cells and this allowed us to monitor the kinetics of B cell development and to test for factors that influence this process.

The Cre/*LoxP* technique is most often used for the conditional deletion of floxed genes. For these loss-of-function approaches, the efficiency of Cre activity is particularly important, as a low frequency of gene deletion may not result in a detectable phenotype. On the other hand, for the activation of a gene with a selective advantage (gain-of-function), the tightness of Cre regulation is more important than its efficiency, and here the MerCreMer recombinase can be employed successfully for developmental studies in the mouse. Indeed, the activity of MerCreMer is extremely well regulated as none of the many pro-B cells that are generated daily in the imb-1 mouse mature in the absence of tamoxifen. This is in line with an earlier study using our mb-1MerCreMer mouse model to induce a conditional RAG1 allele and subsequently a wave of B cell development including B-1 B cells (48). Thus, we argue that the double fusion Cre enzyme MerCreMer, is one of the most tightly regulated Cre enzymes available.

The oral administration of a single dose of 6 mg of tamoxifen to either imb-1 or imb-1/B1-8H mice induces a homogeneous and transient wave of developing B cells that allowed us to study the kinetics of B cell development. The first IgM^+^ B cells appeared between days 5 and 6 p.i. in the BM. This took longer than the 2 days reported for B cell maturation in the BM of BALB/c mice measured by BrdU labeling (75). The reason for this delay may be due to the fact that in tamoxifen-treated mice, it takes 4 days before the tamoxifen concentration in the blood falls below the threshold of MerCreMer activation (Figure S1A in Supplementary Material). Because the mb-1/EGFP cassette can be repeatedly recombined, pro-B cells with stable Ig-α expression are probably produced only after day 4 in these mice. As soon as immature B cells appeared in the BM and the blood of imb-1 mice, they were also found in the spleen, suggesting that the completion of V(D)J recombination and the export of B cells from the BM to the spleen is a rapid process. It is also possible, and we have no way to exclude, that a fraction of B cell development takes place directly in the spleen. Consistent with previous findings, the first immigrant B cells in the spleen are IgM^high^ T1-B cells that are negative for both the CD23 and the CD21 markers (21). However, we also detect an intermediate CD23^+^CD21^−^ B cell population that appears prior to the generation of the CD23^+^CD21^+^ T2-B and follicular B cells (Figures 3A,C, d5–d6; Figure S1B in Supplementary Material, d5–d6) (20). Thus, our data suggest that splenic B cell maturation proceeds first *via* the upregulation of CD23 and than with that of CD21. Interestingly, a study by the Allman group has previously identified a population of newly formed immature B cells in the BM, which was CD93^+^CD23^+^CD21^−^ and thus resembled later transitional stages in the spleen. However, the developmental kinetics and the renewal rate of this population resembled those of immature B cells in the BM. The authors of this study have thus concluded that newly immigrant populations from the BM are heterogeneous and comprise mature and semi-mature B cells (76). Since the imb-1 and imb-1/B1-8 mice are devoid of peripheral B cells prior to the tamoxifen-induced B cell development, it is possible that such semi-mature B cells are more prominently represented in the spleens of these mice compared to mice with an intact B cell development.

In imb-1 mice, the development of T1-B into T2-B cells is a rapid process that occurs in 3–4 days, whereas the development of T2-B cells into follicular or MZ B cells takes roughly 10–20 days. It is not clear why the final maturation of B cells in the spleen takes so long, but it is likely that T2 B-cells require additional signals for their maturation. This notion is supported by the fact that mutant mice lacking elements of the BCR signaling pathways are often arrested at the T2-B cell stage (77). BrdU-labeling experiments indicate that most of the newly emigrant B cells have a short life span (3–5 days) and that only a few of the transitional B cells enter the long-lived B cell pool (17, 78). This suggests that many immature and transitional B cells are primed for negative selection upon engagement of their BCR by self-antigens. Indeed, in mouse models of tolerance the transitional B cells show a rapid antigen-induced apoptosis (79–82). Furthermore, *in vitro* studies with different immature B cell lines or with purified transitional splenic B cells indicate that the main response upon exposure to anti-BCR antibodies is apoptosis (59, 83). It thus came as a surprise that T1-B cells exposed to anti-IgM reagents in imb-1 mice do not die but develop into T2-B cells upregulating the pro-survival factor Bcl-2 (Figures 4 and 5A). Moreover, *in vivo* treatment with anti-IgM resulted in a phenotype of IgM, IgD, and CD23 expression similar to that of mature B cells in wild-type mice (Figure S3 in Supplementary Material). This finding is reminiscent of that shown by the expression of Nur-77 in splenic B cells (84) and suggests that maturation of T1-B cells into T2 and follicular B cells require some level of BCR engagement.

However, one should keep in mind that anti-BCR antibodies are not stimulating the BCR in the same way as cognate antigen (85). Moreover, goat anti-IgM was used for these studies. Thus, we cannot exclude the possibility that after anti-IgM injection into mice, the T1 B cells presented goat Ig-derived peptides to T cells, which then promoted B cell survival and maturation *via* T cell help (86, 87). However, we find this unlikely given that we injected F(ab′)_2_ Ab fragments without adjuvant, and that the changes in B cell maturation were already visible two days after injection, not only in imb-1 but also in Rag2^−/−^γ_c_^−/−^ mice, injected with imb-1-derived B cells (Figure 4; Figure S3 in Supplementary Material). Furthermore, due to low expression of the BCR on the surface of imb-1 B cells, the BCR nanoislands might not be formed correctly, resulting in altered response to BCR engagement (88, 89). Furthermore, the survival of the BCR-stimulated T2-B cells in the imb-1 mice was strictly dependent on the BAFF signaling system whereas non-stimulated naïve T1-B and T2-B cells could survive in these mice for some time without BAFF. We are aware of the fact that imb-1 mice are lymphopenic where the few generated B cells do not compete for limiting growth factors as is probably the case for newly generated B cells in wild-type mice (39, 65, 90, 91). A survival of auto-reactive T cell clones in the thymus has also been observed in a lymphopenic mouse model (52). However, we think that the imb-1 model may help to better characterize the survival niches of B cells of defined developmental stages *in vivo*. Clearly, in the imb-1 mouse, the stimulated T1-B cells require more pro-survival signals such as BAFF than non-stimulated T2-B cells, similar to what has previously been observed for the anergic anti-hen egg lysozyme B cells (40, 63). Only the stimulated T2-B cells, however, displayed an upregulation of the pro-survival factor Bcl-2, indicating that the NF-κB signaling pathway was activated in these cells. Indeed, genetic and biochemical studies have shown that the canonical and non-canonical NF-κB signaling pathways play an essential role for the survival and further development of T2-B cells (36, 61, 92). It was recently suggested that the signals from the BCR and the BAFF-R cooperate to ensure a prolonged NF-κB activation and, thus, the survival of stimulated B cells (62). Furthermore, it has been shown that the overexpression of either Bcl-2 or Bcl-xL rehabilitates the survival of BAFF-R deficient B cells, suggesting that BAFF rescues B cells from apoptosis (31, 38, 93). However, our studies of *in vitro* cultured T1-B cells from imb-1 mice suggest that BAFF is not the only factor controlling the survival of transitional B cell *in vivo*. According to Sandel and Monroe (55), the site of antigen encounter is important for the decision whether immature B cells are rescued or undergo apoptosis. Therefore, the cellular BM microenvironment protects immature B cells from apoptosis by inducing RAG re-expression and subsequent receptor editing whereas splenic microenvironment does not prevent the rapid demise of stimulated transitional B cells resulting in their negative selection. In the BM, CD90^lo^ cells, which have later been identified as basophils (94), seem to provide the rescuing signal to immature B cells. Interestingly, basophils (and also mast cells) have been attributed with the production of high levels of BAFF, IL-4, IL-6, IL-13, and other cytokines, thereby contributing to the survival niche of immature but also mature activated B cells *in vivo*. Basophils have even been reported to home to sites of inflammation in the periphery thus supporting activated B cells and plasma cells [reviewed in Ref. (95)]. It is thus feasible that, due to lymphopenia in imb-1 mice at the start of the experiments, there is an altered splenic microenvironment, where basophils, mast cells, and their products contribute to the enhanced survival of anti-IgM-treated T1 B cells *in vivo*. Furthermore, not only *in vitro* but also in an *in vivo* situation, stimulation through CD40 certainly plays an important role in B cell activation. From the results shown in Figure S3 in Supplementary Material one could assume, however, that CD40 ligand (L) does not account for the survival of the T1 B cells after anti-IgM stimulation *in vivo* since T1 B cells stimulated with anti-IgM still survive in the transfer experiments into Rag2^−/−^γc^−/−^ mice, which lack T cells and therefore CD40L. However, other cell types, including basophils and mast cells, can express CD40L, thus mimicking “T-cell help” (95) and might therefore play an important role in the *in vivo* survival, in addition to BAFF. Clearly more remains to be learned about the signals that control the selection of B cells (*in vitro* and) *in vivo*, and the imb-1 mouse strain represents a useful model in which to study these events in more detail.

Finally, the imb-1 and RERT/EGFP models can be employed in studies of BCR signaling in lymphoma and leukemogenesis. Many B cell lymphomas are dependent on BCR signaling (96), such as, e.g., chronic lymphocytic leukemia (CLL). Combining the imb-1 or RERT/EGFP mouse lines with transgenic Tcl-1 lines, the latter serving as model for CLL (97), would result in generation of B cells either with low or with nearly physiological BCR surface expression and would thus allow studying the influence of BCR expression dosage on CLL development.

## Materials and Methods

### Generation of Targeting Vector and Targeted ES Cell Clones

Two genomic clones containing the complete mouse *mb-1* locus from the BALB/c strain (98) were kindly provided by Dr. N. Sakaguchi (Kumamoto, Japan) and were used for the generation of the mb-1 MerCreMer targeting vector in order to replace exons 1 and 2 of the *mb-1* locus. The Cre cDNA was derived from the pBlueiCre plasmid kindly provided by R. Sprengel (Max Planck Institute for Medical Research, Heidelberg, Germany) (99). A 3-kb cDNA encoding for MerCreMer was recovered from the pAn MerCreMer vector, containing the cDNA of MerCreMer under the human β-actin promoter and subcloned in the pBSmb-1 backbone, which encodes the BALB/c genomic *mb-1* promoter and the short arm of homology of the *mb-1* locus. The ATG codon derived from MerCreMer is in-frame with the ATG from exon1 of the *mb-1* gene. In addition, the splice donor site from the mb-1 exon 3 was introduced by oligonucleotides after the stop codon of MerCreMer. The E-mu enhancer was introduced as a blunted 990 bp XbaI fragment 5′ from the *mb-1* promoter. The 9.6 kb genomic long arm of homology beginning within intron 3, as well as a Neo^r^ cassette flanked by two Frt sites in the same direction, was cloned in a last step to give the targeting construct. The generation of the reporter mEGFP/mb-1^inv^ vector was described before (47).

BALB/c ES cells (1 × 10^7^) (100) were electroporated in 900 µl transfection buffer (20 mM HEPES, pH 7.0; 137 mM NaCl; 5 mM KCl; 0.7 mM Na_2_HPO_4_; 6 mM glucose; 0.1 mM 2-β-ME) with 20 µg linearized vector at 240 v and 475 μF. ES cells were cultured in DMEM selection medium containing 10% FCS, l-glutamine, sodium pyruvate, penicillin/streptomycin, and G418 (320 µg/ml). Three clones out of 480 gave the expected bands for the targeted allele by Southern blot analysis. Digestion with *EcoR*I results in a 4.1 kb fragment for mb-1 MerCreMer and a 7.6 kb fragment for both mEGFP/mb-1^inv^ and mb-1/mEGFP^inv^, compared with a 6.4 kb fragment from BALB/c genomic DNA. The neo cassette was deleted by transient expression of the enhanced (Flpe) recombinase kindly provided by Dr. F. Stewart (Dresden, Germany) (101). Two independent clones were injected into C57BL/6J blastocysts at the transgene facility of the Max-Planck-Institute of Immunobiology (Freiburg, Germany). Five chimeric mice were obtained, and only one transmitted the mutation to the germ line.

### Mouse Experiments

The reporter mice mEGFP/mb-1^inv^ and mb-1/mEGFP^inv^ have been described (47). Intercrossing of mb-1MerCreMer mice to mEGFP/mb-1^inv^ mice gave rise to the imb-1 strain, and crossing mb-1MerCreMer to mb-1/mEGFP^inv^ resulted in the cmb-1 strain. The B1-8H mice (57) harboring a VDJ transgene derived from the (4-hydroxy-3-nitro-phenyl acetyl) (NP)-binding antibody B1-8 (102) at the VH locus, were crossed to imb-1 and cmb-1. The RERT mouse strain (70) was kindly provided by M. Barbacid (Madrid, Spain) (71). The crossing of the RERT with the mEGFP/mb-1^inv^ strain generated the RERT/EGFP mouse strain, schematically depicted in Figure S4B in Supplementary Material. The B1-8H and BAFF-R^−/−^ mouse strain was obtained from K. Rajewsky (38, 57). Mating of the BAFF-R^−/−^ mouse with the imb-1 mouse resulted in the imb-1/BAFF-R^−/−^ mouse strain. Mice used throughout this study were 6–8 weeks old. All animal studies were carried out in accordance with the German Animal Welfare Act, having been reviewed by the regional council and approved under license #G-09/103. All mice were maintained in a barrier mouse facility, or under specific pathogen-free conditions at the animal facility of the MPI-IE.

### Southern Blot and PCR Analysis

A 170-bp genomic *mb-1* fragment located 2 kb 5′ of the *mb-1* promoter (103) was used as an external probe to discriminate between wild-type (7.6 kb) and the MerCreMer targeted allele (4.1 kb) when hybridized to *Eco*RI*-*digested genomic DNA. Screening for the reporter mice has been described earlier (47). PCR screening of genomic DNA derived from tails of the different transgenic mice used in the experiments was done as follows: for MerCreMer detection, an hCre PCR was used with the following primers hCredir: CCCTGTGGATGCCACCTC and hCrerev: GTCCTGGCATCTGTCAGAG. Ta = 58°C; Tex = 1 min and 30 cycles give a 450-bp product. In addition, an EGFP PCR was designed to detect the insertion of mEGFP/mb-1^inv^ or mb-1/mEGFP^inv^ in the reporter strains. Two primers were designed GFPdir; GGTGGTGCCCATCCTGGTCG and GFPrev: GTACAGCTCGTCCATGCCGAG. A 700-bp product is detected under the same conditions as in the above-mentioned PCR. Homozygous mice were identified by the lack of B lymphocytes in peripheral blood caused by the Ig-α deficiency.

### Tamoxifen Treatment of Mice

Tamoxifen citrate^®^ tablets 30 mg (AstraZeneca) were emulsified in clinoleic^®^ 20% (Baxter), a mixture of olive and soybean oil in a 5:1 ratio. In all experiments, mice were treated 1× orally with 6 mg the suspension, by gavage with the help of a curved needle. Mice were then analyzed at different times p.i.

### Analysis of Mice With Inducible B Cell Development

The absence of B cells in imb-1 and RERT/EGFP mice before induction with tamoxifen citrate was verified by FACS analysis of peripheral blood. The mice were bled again at day 5 p.i. and monitored for B cell development in the periphery. The mice with comparable relative B cell percentage in their peripheral blood were chosen for each experiment.

### Treatment of Mice

imb-1 or RERT/EGFP mice were injected i.p. at day 5 p.i., with 100 µg goat anti-mouse IgM heavy chain antibodies [polyclonal IgG F(ab′)_2_; #115-006-075] (Jackson ImmunoResearch laboratories). Another goat anti-mouse IgM F(ab′)_2_ fragment (endotoxin low) was applied in the same studies to ensure for reproducibility (Southern biotechnology cat. #1022-14). Untreated mice received polyclonal goat IgG, F(ab′)_2_ fragment (cat# 005-000-006) (Jackson ImmunoResearch laboratories). TACI-Ig was purchased from (R&D). The mice were injected i.p. three times at days 0, 3, and 5 p.i. with 50 µg per mouse TACI-Ig (Atacicept, Merck) and were monitored at day 7 p.i.

### Generation of the J558 MerCreMer/loxP-Stop-loxP mEGFP Reporter Cell Line

The myeloma cell line J558L μm3-11 MerCreMer/*loxP*-Stop-*loxP* mEGFP (referred to as J558; MCM/loxEGFP) reporter cell line carries a Cre-reporter vector with a *loxP*-Stop-*loxP* EGFP cassette coding for an mEGFP, expressed under the control of the human β*-actin* promoter. In the 5′ region of the vector a floxed DNA cassette bearing a Stop codon and a polyA sequence prevents the expression of EGFP. Cre-mediated deletion of the floxed Stop sequence results in EGFP expression and can be monitored by FACScan analysis. The cell line was generated as follows. Two MCM targeting vectors were linearized and transfected into the J558L/*loxP*-stop-*loxP* EGFP reporter cell line. For this the reporter cells growing at the exponential phase were harvested by centrifugation (Varifuge 3.0 F, 339 × *g*, at 4°C for 5 min) and washed twice with PBS and 1 × 10^7^ cells were resuspended in 300 µl of RPMI medium and transferred into an electroporation cuvette (EquiBio ECU 104). Linearized plasmid DNA (10 µg) purified with the Qiagen Maxi Kit was added and the cells were incubated on ice for 5 min. The cells were electroporated using a BioRad Gene pulser II at 260 V, 960 μF, after which the samples were maintained on ice for another 10 min. A “mock” control containing the same cell number was subjected to electroporation in the absence of DNA. The electroporated cells were resuspended in 48 ml RPMI medium, plated into two 24-well plates (Greiner) at 1 ml/well and incubated at 37°C and 5% CO2. The medium was replaced after 2 days with fresh medium containing the desired selection factor (G418 or hygromycin).

### Preparation of Cell Suspension From Lymphoid Organs

Single cell suspensions from BM, spleen, and blood were prepared as described (103).

### Antibodies for FACS Analysis

For each sample 1–2 × 10^6^ cells were incubated with various combinations of antibodies as indicated. The antibodies for lymphocyte staining were against the following mouse antigens: B220 (clone RA3-6B2), CD19 (clone 1D3), CD21 (clone 7G6), CD23 (clone B3B4), IgD (clone 11-26), Bcl-2 (clone 3F11), purchased from BD (Becton Dickinson). Biotinylated Abs were detected by Streptavidin-PerCp (BD). Anti-mouse IgM was from Jackson ImmunoResearch Laboratories. Anti-mouse CD93 (C1qRp; clone AA4.1), MHC class II (I-A/I-E) (clone M5/114.15.2), CD62L (clone MEL-14), CD24 (clone M1/69), CD40 (clone 1C10) antibodies were from (eBioscience), and anti-CD40 antibodies (clone 3/23) were from (BD). In the mouse experiments, the dead cells were excluded based on FSC/SSC. In the *in vitro* experiments, 7-aminoantinomycin D (7AAD) was used to exclude dead cells and was purchased from (SouthernBiotech). Four-color flow cytometry was performed on a FACSCalibur™ flow cytometer (BD). Flow cytometric profiles were analyzed using CELLQuest™ software (BD) and FlowJo (Tree Star). Prior to all the stainings, the cells were incubated with an anti-Fc receptor antibody (clone 2.4G2) to block unspecific binding.

### Intracellular Staining

For each sample 1–2 × 10^6^ cells were washed in PBS and then fixed in a 2% formaldehyde solution for 15 min at room temperature. The cells were washed two times in PBS and then stained for 15 min at room temperature with anti-Bcl-2 (Clone 3F11; BD) or anti-Bcl-xL (Clone H-5, Santa Cruz Biotechnology) in 0.05% saponin in PBS.

### Cell Sorting, Transfer, and *In Vitro* Stimulation

Transitional 1-B cells from imb-1 mice were sorted by negative selection using CD43 and Thy1.2 antibodies to exclude non-B cells. T1-B cells from RERT/EGFP mice were sorted based on EGFP expression. These cells closely resemble imb-1-derived T1-B cells due to their expression of one copy of the mb-1 allele, whereas EGFP is expressed from the second mb-1 allele and thus serves as a reporter. To exclude CD23^+^ population, the cells were further stained with an anti-CD23 antibody (clone B3B4; BD). The sorted EGFP^+^CD23^−^ T1-B cells (purity ≈ 98%; Figure S3B in Supplementary Material) were immediately transferred i.v. into Rag2^−/−^γ_c_^−/−^ mice and either left untreated or treated with F(ab′)_2_ fragment of anti-IgM 3 h after transfer of EGFP positive cells.

For *in vitro* stimulation experiments, soluble human recombinant BAFF (R&D) was applied at 75 ng/ml; anti-CD40 (FGK45) was a gift from the late Ton Rolink and was applied at 10 µg/ml; anti-IgM [polyclonal IgG F(ab′)_2_; #115-006-075] was applied at 10 µg/ml.

### Statistical Analysis

Statistical testing was performed using one-sample *t*-test unpaired two-tailed Student’s *t-*test (with *n* = five mice per group) was carried out using Prism 4 software (GraphPad Software Inc.).

## Ethics Statement

Mice used throughout this study were 6–8 weeks old. All animal studies were carried out in accordance with the German Animal Welfare Act, having been reviewed by the regional council and approved under license #G-09/103. All mice were maintained in a barrier mouse facility, or under specific pathogen-free conditions at the animal facility of the MPI-IE.

## Author Contributions

EH, RP, and MR designed the study. EH, EL-Z, and MD conducted the experiments. MR, EH, RP, and EL-Z wrote the manuscript.

## Conflict of Interest Statement

The authors declare that the research was conducted in the absence of any commercial or financial relationships that could be construed as a potential conflict of interest.
