# Supplementary material for: Conditional Selection of B Cells in Mice With an Inducible B Cell Development
Source: Front Immunol. 2018 Aug 6;9:1806. doi: 10.3389/fimmu.2018.01806 (PMC6087743; doi:10.3389/fimmu.2018.01806)
Supplement: Supplementary file 1 [file Data_Sheet_1.pdf]

Conditional selection of B cells in mice with an inducible B cell development

Elias Hobeika[*1,2], Marcel Dautzenberg[1], Ella Levit-Zerdoun[4, 5], Roberta Pelanda[3] and Michael Reth[*1]

[1]Centre for Biological Signaling Studies (BIOSS); Biology III, Faculty of Biology, Albert-Ludwigs-University of Freiburg, 79104 Freiburg, Germany; Max Planck Institute of Immunobiology and Epigenetics, 79108 Freiburg, Germany,

[2]present address: Dep. of Tumor Immunology, Institute of Immunology, Ulm University Hospital D-89081 Ulm, Germany

[3]present address: Department of Immunology and Microbiology, University of Colorado Denver School of Medicine, Aurora, CO 80045, USA; Department of Biomedical Research, National Jewish Health, Denver, CO 80206, USA

[4]Max Planck Institute of Immunobiology and Epigenetics, 79108 Freiburg, Germany; Department of Molecular Immunology, Biology III, Faculty of Biology, Albert-Ludwigs-University Freiburg, 79104 Freiburg, Germany; International Max Planck Research School for Molecular and Cellular Biology, 79108 Freiburg, Germany

[5]present address: German Cancer Consortium (DKTK) partner site Freiburg, German Cancer Center (DKFZ), Heidelberg, Institute of Molecular Medicine and Cell Research, 79104 Freiburg, Germany

***Correspondence:**
Michael Reth: Michael.Reth@bioss.uni-freiburg.de

Elias Hobeika: elias.hobeika@uni-ulm.de

Supplementary Figure 1

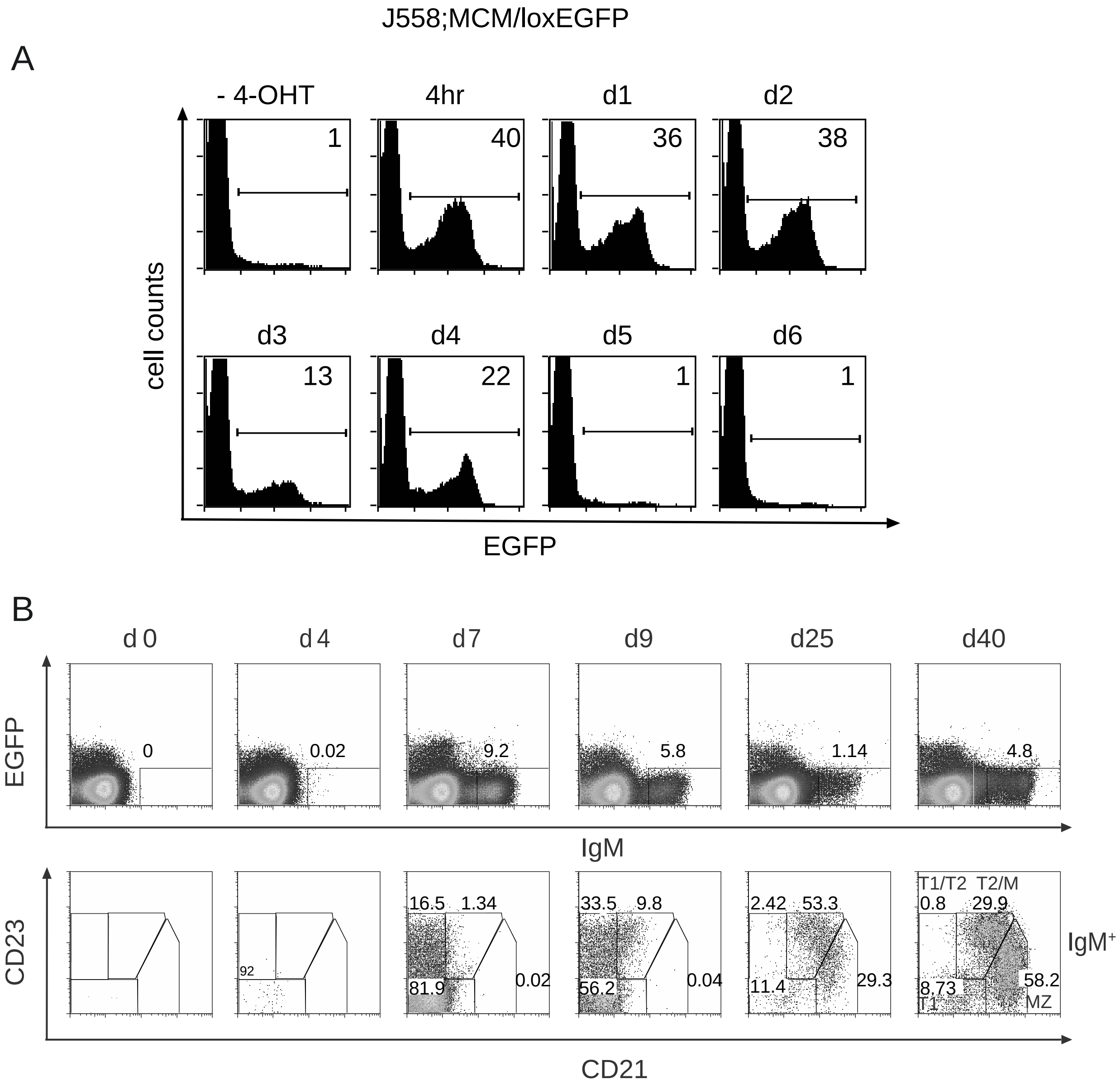

Supplementary Figure 1. (A) Tamoxifen-citrate is active up to 4 days in the mice. Imb-1/B1-8H mice were treated with a single dose of tamoxifen citrate and each mouse was bled to obtain serum at several time points, indicated above the panels (4 h – d 6 p.i.). Each sample of serum was added to cultures of J558 MerCreMer/stop-lox-stop mEGFP reporter cell line and the cells were analysed for EGFP expression after 48 h. Data from 2 independent experiments with two mice at each time point are shown. **(B) Transitional B cells express CD23 before CD21.** FACS analysis of CD23 and CD21 expression of the IgM$^+$ B lymphocyte populations derived from the spleens of imb-1/B1-8 mice before (d0) or at indicated time points after a single treatment with tamoxifen. Representative data from 2 independent experiments with total five mice at each time point are shown

Supplementary Figure 2

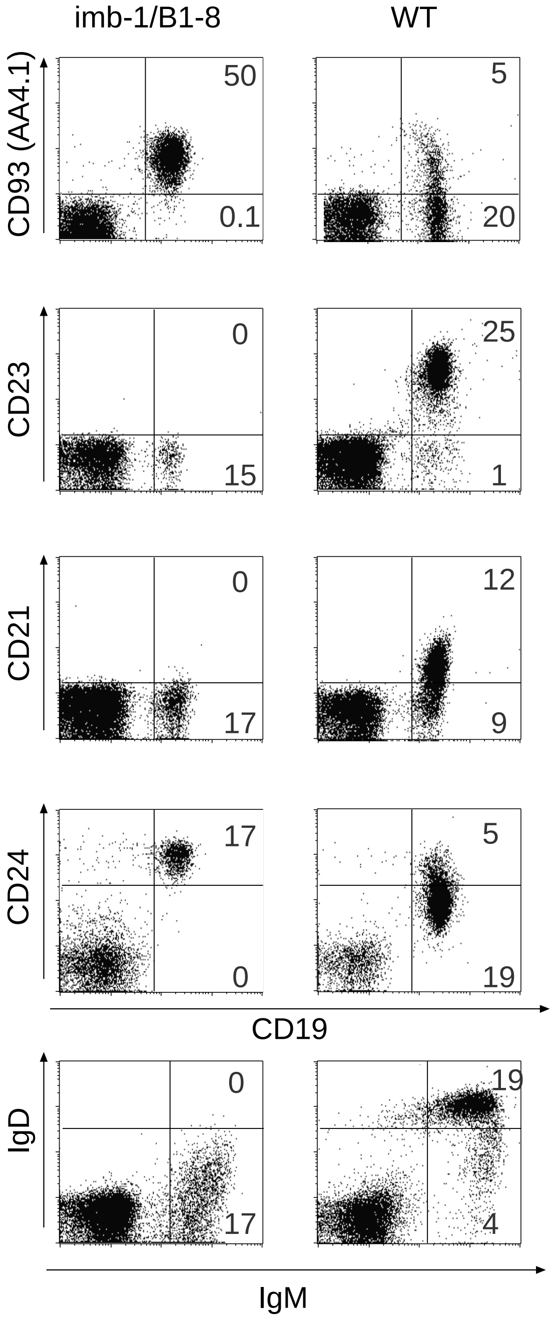

Supplementary Figure 2. Phenotypic characterization of transitional B cells from day 5 of induced imb-1 mice. Splenic B cells from wild-type BALB/c (right panels) and from day 5 p.i. imb-1/B1-8H mice (left panels) were isolated and stained with antibodies against CD19 and either CD93

(AA4.1), CD23, CD21 or CD24 (HSA). Lower panel shows the analysis of a IgM versus IgD staining. Representative data from 5 independent experiments with two mice are shown.

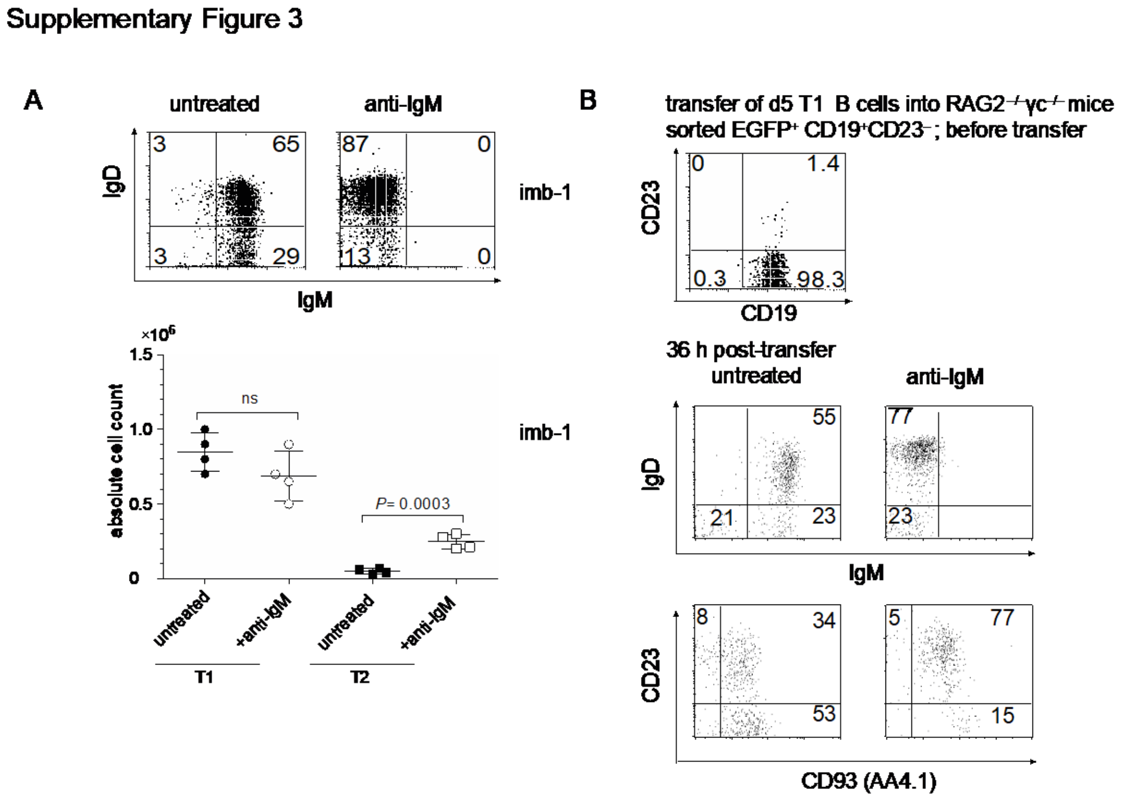

Supplementary Figure 3. Anti-IgM treatment of sorted T1-B cells transferred into immunodeficient mice. (A) Upper panel: Mice from the imb-1 strain were induced with tamoxifen and left untreated or received anti-IgM at d5 p.i. The expression of IgM versus IgD on CD19$^+$ B lymphocytes collected at day 7 p.i. is shown. Representative data from 4 independent experiments with 2 mice each are shown. Lower panel: statistical analysis of the absolute cell count of T1 (CD19$^+$CD23$^-$) and T2 (CD19$^+$CD23$^+$) B cells from untreated or anti-IgM treated mice at day 7 p.i. Each dot indicates an individual animal; p-Values were obtained using a two-tailed Student's t-test; ns= not significant.(B) Upper row: Sorted transitional EGFP$^+$ B cells (d5 p.i.; CD19$^+$CD23$^-$) derived from induced RERT/EGFP mice (see below in Supplementary Figure 4) were transferred into Rag2$^{-/-}\gamma_c^{-/-}$ mice, which were either left untreated or received anti-IgM 3 h post-transfer. The spleens of the host mice were analyzed for B cells 36 h post-transfer. Dot plots of IgM versus IgD (middle row) and CD93 (AA4.1) against CD23 (bottom row) expression on EGFP$^+$ B cells collected from untreated (left panel) or from anti-IgM treated mice (right panel) are shown. The numbers indicate the percentage of the different B cell populations. Representative data from 3 independent experiments with two mice are shown.

Supplementary Figure 4

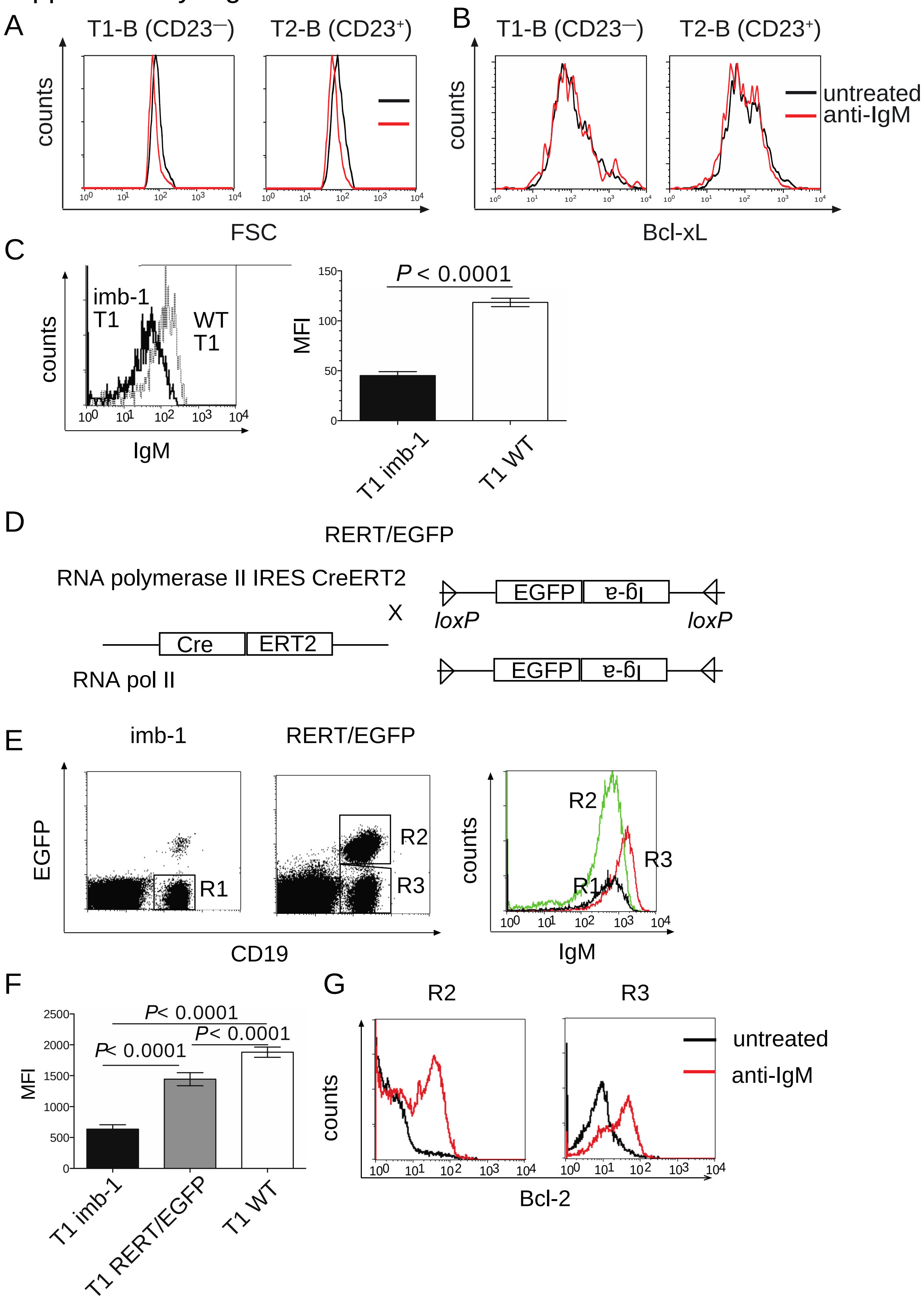

Supplementary Figure 4. Levels of the IgM-BCR on T1-B cells derived from imb-1 and RERT/EGFP mice. (A) Histograms of forward scatter (FSC) of splenic B cells derived from $CD21^{-}CD23^{-}IgM^{high}IgD^{low}$ T1 (left panel) and $CD21^{+}CD23^{+}IgM^{high}IgD^{high}$ T2 (right panel) B cells. B cells from untreated mice, black line; from anti-IgM treated mice, red line. The cells were analysed at day 7 p.i. (2 days after anti-IgM injection). Representative data from 5 independent experiments with two mice are shown. (B) Histograms of forward scatter Bcl-xL expression of splenic B cells derived from $CD21^{-}CD23^{-}IgM^{high}IgD^{low}$ T1 (left panel) and $CD21^{+}CD23^{+}IgM^{high}IgD^{high}$ T2 (right panel) B cells. B cells from untreated mice, black line; from anti-IgM treated mice, red line. The cells were analysed at day 7 p.i. (2 days after anti-IgM injection). Representative data from 5 independent experiments with two mice are shown. (C) IgM expression levels on splenic T1-B cells derived from imb-1 compared to wild-type mice. imb-1, dark line and wild-type, dashed line. Right panel: median fluorescence intensity (MFI) of *n*=5 mice; shown are mean±SD bar graphs. The P value was obtained using a two-tailed Student's *t*-test. (D) Schematic representation of the Cre-ER^{T2} and the mb-1 alleles of the RERT/EGFP mouse strain. The RERT/EGFP mouse strain is the result of a mating between the RNA polymerase II Cre-ER^{T2} and the mb-1 EGFP/Ig-α mice strains. The homozygosity at the *mb-1* locus leads to Ig-α deficiency. Tamoxifen application to these mice induces Cre activity, *loxP* recombination and consequently Ig-α expression. (E) Comparison of IgM expression in the T1-B cell populations derived from induced imb-1 and RERT/EGFP mice. Middle panel: a CD19 versus EGFP dot plot of lymphocytes derived from RERT/EGFP mice at day 5 p.i. Two T1-B cell populations, R2, in which B cells are $EGFP^{+}$ and express Ig-α from only one allele, and R3, in which B cells are $EGFP^{-}$ and express Ig-α from both alleles. These $CD19^{+}$ $EGFP^{+}$ (R2) and $CD19^{+}$ $EGFP^{-}$ (R3) cells were simultaneously stained for surface IgM expression. $EGFP^{+}$ (green line, R2), $EGFP^{-}$ (red line, R3), imb-1 (black line, R1). (F) Median fluorescence intensity (MFI) represented as mean±SD bar graphs of *n*=5 mice. imb-1-derived T1-B cells carry one copy of *mb-1* (from R1), RERT/EGFP-derived EGFP− T1-B cells carry two copies of *mb-1* (from R3), wild-type (WT) B cells carry two WT *mb-1* alleles (gate not shown). P values were obtained using a two-tailed Student's *t*-test. (G) Comparison of Bcl-2 expression in transitional B cells from the induced RERT/EGFP mice. Overlay histogram plots comparing intracellular Bcl-2 expression in $EGFP^{+}$ R2 (left panel) and $EGFP^{-}$ R3 (right panel) in transitional B cells derived from RERT/GFP mice at day 7 p.i., either untreated (black line) or treated with anti-IgM at day 5 (red line). Data are representative for at least 5 independent experiments.

Supplementary Figure 5

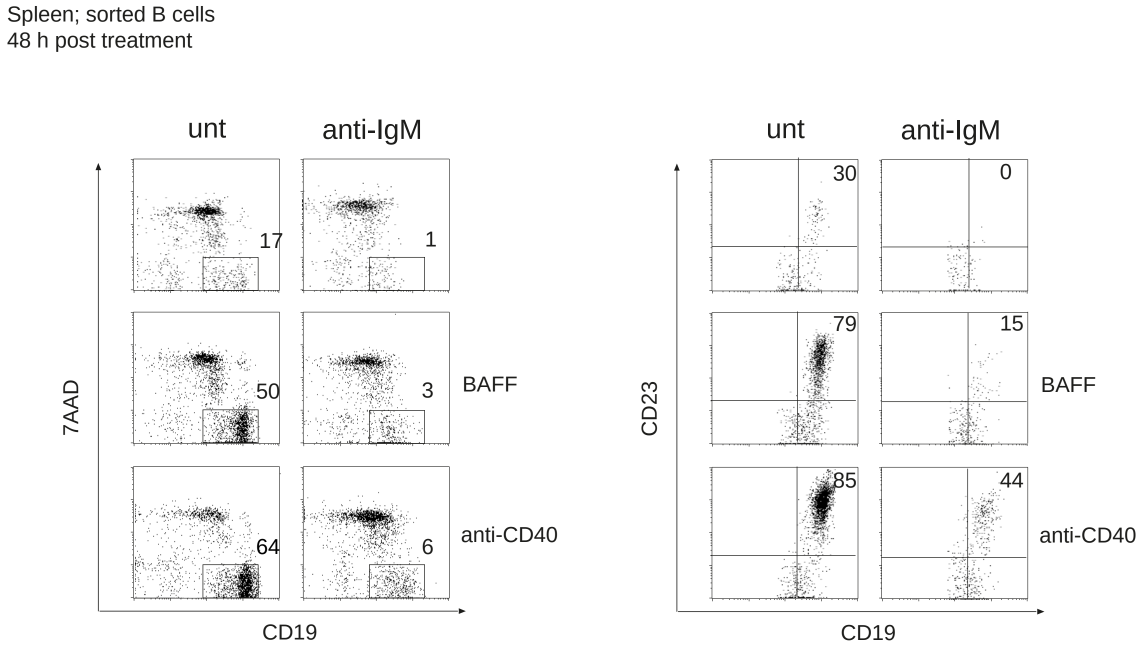

Supplementary Figure 5. Anti-IgM treatment of T1 B cells *ex vivo* (48 h). (A) imb-1-derived splenocytes were sorted at day 6 p.i using CD43 and Thy1.2 antibodies to exclude non-B cells. Identical cell numbers were cultured *in vitro* in the absence or presence of anti-IgM (10 µg/ml) and/or human recombinant BAFF (75 ng/ml) and anti-CD40 antibodies (10 µg/ml) for 48 hours. The viable cells were selected by gating on the $CD19^{+}7AAD^{-}$ population and further represented as CD19 versus CD23 dot plots. The numbers in quadrants indicate relative cell numbers acquired after analysis of 10^5 total events per sample
